# Health Consequences of Child Marriage in Africa

**DOI:** 10.3201/eid1211.060510

**Published:** 2006-11

**Authors:** Nawal M. Nour

**Affiliations:** * Brigham and Women’s Hospital, Boston, Massachussetts, USA

**Keywords:** Child marriage, early marriage, maternal mortality, HIV/AIDS, fistula, perspective

## Abstract

Despite international agreements and national laws, marriage of girls <18 years of age is common worldwide and affects millions. Child marriage is a human rights violation that prevents girls from obtaining an education, enjoying optimal health, bonding with others their own age, maturing, and ultimately choosing their own life partners. Child marriage is driven by poverty and has many effects on girls' health: increased risk for sexually transmitted diseases, cervical cancer, malaria, death during childbirth, and obstetric fistulas. Girls' offspring are at increased risk for premature birth and death as neonates, infants, or children. To stop child marriage, policies and programs must educate communities, raise awareness, engage local and religious leaders, involve parents, and empower girls through education and employment.

## Abstract 2

Comprehensive, multifaceted policies are needed to end child marriage and protect girls and their offspring.

Awareness of reproductive health issues in developing nations is growing. Critical issues are the high prevalence of HIV/AIDS among young people; childbearing by young girls, which can lead to obstetric fistulas and death of the mother; and child marriage.

Child marriage, defined as marriage of a child <18 years of age, is an ancient, worldwide custom. Other terms applied to child marriage include "early marriage" and "child brides." Early marriage is vague and does not necessarily refer to children. Moreover, what is early for one person may be late for another. Child bride seems to glorify the process, implying a celebration and a bride who is happy to start a loving union with her spouse. But for the most part, girl brides do not know—and may have never met—their groom.

In 2002, ≈52 million girls <18 years of age were married. With ≈25,000 girls <18 years being married each day, an estimated 100 million will be married by 2012 (*1*). Child marriages occur most frequently in South Asia, where 48% of women aged 15–24 have been married before the age of 18; these figures are 42% for Africa and 29% for Latin America and the Caribbean (*2*).

Although the definition of child marriage includes boys, most children married at <18 years of age are girls. For example, in Mali the girl:boy ratio of marriage before age 18 is 72:1; in Kenya, 21:1; and even in the United States, 8:1 (*3**–**5*). We therefore focus on the social and health consequences of child marriage for girls. And although we focus on African countries, similar arguments over what drives child marriages, how they affect girls, and how to stop them may be applied to other continents.

## United Nations Efforts and National Laws

Since 1948, the United Nations and other international agencies have attempted to stop child marriage. Article 16 of the Universal Declaration of Human Rights states that persons must be at "full age" when married and that marriage should be entered into "freely" and with "full consent." In other words, any country that allows child marriage is committing a violation of human rights (*6*). Articles 1, 2, and 3 of the 1962 Convention of Consent to Marriage, Minimum Age for Marriage, and Registration of Marriages require that countries establish a minimum age for marriage and that all marriages be registered (*7*). Article 16 of the 1979 Convention on the Elimination of All Forms of Discrimination against Women requires minimum ages for marriage to be specified and says that child marriages are illegal (*8*). However, not until 1989, at the Convention on the Rights of the Child, did international law define children as persons <18 years of age (Article 1) (*9*). In 1994, the International Conference on Population and Development stated that the minimum age of marriage should be raised and enforced, all forms of coercion and discrimination should be eliminated, marriage should be entered into with free consent and as equal partners, and the education and employment of girls should be encouraged (Principle 9, Action 4.18, Action 5.5) (*10*).

In many countries, the legal age for marriage is 18, yet some governments enforce these laws loosely. For example, the percentage of girls married before age 18 in Niger is 77%, in Chad 71%, in Mali 63%, in Cameroon 61%, and in Mozambique 57% (*1*). In parts of Ethiopia, 50% of girls are married before the age of 15, and in Mali, 39%. Some marriages even occur at birth; in such instances, the girl is sent to her husband's home at the age of 7 (*11*).

## Incentives for Perpetuating Child Marriages

Poverty plays a central role in perpetuating child marriage. Parents want to ensure their daughters' financial security; however, daughters are considered an economic burden. Feeding, clothing, and educating girls is costly, and girls will eventually leave the household. A family's only way to recover its investment in a daughter may be to have her married in exchange for a dowry. In some countries, the dowry decreases as the girl gets older, which may tempt parents to have their daughters married at younger ages. These are not necessarily heartless parents but, rather, parents who are surviving under heartless conditions. Additionally, child marriages form new alliances between tribes, clans, and villages; reinforce social ties; and stabilize vital social status.

Parents worry about ensuring their daughters' virginity and chastity. Child marriage is also seen as a protective mechanism against premarital sexual activity, unintended pregnancies, and sexually transmitted diseases (STDs). The latter concern is even greater in this era of HIV/AIDS.

Girls who marry young tend to be from poor families and to have low levels of education. If they marry men outside their village, they must move away. Coping with the unfamiliar inside and outside the home creates an intensely lonely and isolated life. As these girls assume their new roles as wives and mothers, they also inherit the primary job of domestic worker. Because the husband has paid a hefty dowry, the girl also has immediate pressure to prove her fertility. Girls often embrace their fate and bear children quickly to secure their identity, status, and respect as an adult. As a result, these young girls have high total fertility rates but have missed the opportunities to be children: to play, develop friendships, bond, become educated, and build social skills.

Characteristics of the men who marry young girls are also fairly homogenous. Because men have to pay large dowries for girls, many must work for years to generate enough income. As a result, they are older when they marry, which means that they have little in common to discuss with their young wives except household responsibilities and child rearing. Men also are expected to have had multiple sex partners and to be sexually experienced. Because men are aware of the HIV/AIDS danger, they seek even younger, virginal brides, who are presumably not infected.

## Risk for HIV and Other Sexually Transmitted Diseases

A common belief is that child marriage protects girls from promiscuity and, therefore, disease; the reality is quite different. Married girls are more likely than unmarried girls to become infected with STDs, in particular HIV and human papilloma virus (HPV). In sub-Saharan Africa, girls ages 15–19 years are 2–8 times more likely than boys of the same age to become infected with HIV (*12*). The risk of acquiring HIV from a single act of unprotected vaginal intercourse is 2–3 times greater for women than men (*13*). Globally, the prevalence of HIV infections among women is highest from ages 15 to 24; the risk for men peaks 5–10 years later (*12*).

Marriage by age 20 has become a risk factor for HIV infection for young and adolescent girls (*13*), as has been shown by several studies of African populations (*14**–**16*). A study in Kenya demonstrated that married girls had a 50% higher likelihood than unmarried girls of becoming infected with HIV. This risk was even higher (59%) in Zambia. In Uganda, the HIV prevalence rate for girls 15–19 years of age was higher for married (89%) than single girls (66%); for those 15–29 years of age, HIV prevalence was 28% for married and 15% for single girls. This study noted that the age difference between the men and their wives was a significant HIV risk factor for the wives (*16*). All of these studies showed that girls were being infected by their husbands. A hypothesis relevant to this finding is that a young girl may be physiologically more prone to HIV infection because her vagina is not yet well lined with protective cells and her cervix may be more easily eroded. Risk for HIV transmission is also heightened because hymenal, vaginal, or cervical lacerations increase the transmission rate, and many of these young girls lose their virginity to HIV-infected husbands. Also, STDs such as herpes simplex virus type 2 infection, gonorrhea, or chlamydia enhance girls' vulnerability to HIV (*17**–**19*).

Another study explored why married girls in Kenya and Zambia had a higher risk for HIV infection. This study concluded that because married girls are under intense pressure to prove their fertility, they have more unprotected intercourse. The study also found that husbands were substantially older (5–14 years) than their wives and were 30% more likely than boyfriends of single girls to be HIV infected. Because of their age alone, the husbands had already had numerous sex partners. Additionally, in these areas of Africa, polygamy is common (*20*).

One fundamental difficulty with child marriage is that girls are financially dependent on their husbands and therefore lack the power to make demands upon them. They cannot ask their husbands to get an HIV test; they cannot abstain from intercourse or demand condom use (*20*); they cannot insist that their husbands be monogamous; and ultimately, they cannot leave because they cannot repay their high dowry (*21*). In addition, returning to their parents' home may not be an option because divorce is considered unacceptable and leaving their husbands may have serious implications on the social or tribal ties that were developed during the marriage.

## Cervical Cancer

Child marriage and polygamy play an important role in another deadly disease, cervical cancer. HPV infection has become endemic to sub-Saharan Africa (*22**–**24*). Although many African nations do not have the capacity to adequately or effectively screen for cervical cancer or HPV, the incidence of cervical cancer in Africa is estimated to be extremely high. Common risks for cervical cancer are child marriage, low socioeconomic status, poor access to health care, and husbands who had multiple sex partners. For example, in Mali, cervical cancer is the most common cancer in women, has an age-standardized incidence rate of 24.4 per 100,000, and is the second most common cause of death from cancer (*25*). In a case-control study of 200 participants with and without cervical cancer, among whom the mean age at marriage was 15 years, HPV was detected in 97% of the cases and 40% of the controls. The risk factors identified were child marriage, high parity (>10 children), polygamous husbands (>2 wives), and poor genital hygiene (no tap water available and reuse of sanitary napkins). Another study in Morocco had similar findings (*26*), with cervical cancer risk factors identified as child marriage, high parity, long-term use of oral contraceptives, and poor genital hygiene (control participants bathed more frequently, and case-participants used homemade sanitary napkins more frequently). Other studies have also implicated hygiene as a possible factor (*22**,**27*).

## Children Bearing Children

Pregnancy poses many challenges for young girls. Because pregnancy suppresses the immune system (*28*), pregnant girls are at increased risk of acquiring diseases like malaria. Malaria kills >1 million people each year, 90% of them in Africa. Approximately 25 million pregnant women are exposed to malaria per year, and pregnant women are among the most severely affected by malaria. About 10.5 million become infected during their second or third trimester (*29*), and among these, the mortality rate is ≈50% (*30*). Not only are pregnant women most susceptible to malaria during their first pregnancy (*31*), but they also have higher rates of malaria-related complications (predominantly pulmonary edema and hypoglycemia) and death than do nonpregnant women. Malaria parasite density is significantly higher in pregnant girls <19 years than in pregnant women >19 years (*32*). However, a woman who has had malaria during pregnancy is less susceptible to malaria during subsequent pregnancies, unless the woman is also HIV infected (*31*).

The interaction between HIV and malaria in young married girls is devastating. Rates of coinfection are highest in Central African Republic, Malawi, Mozambique, Zambia, and Zimbabwe, where >90% of the population are exposed to malaria and >10% are HIV positive. HIV-infected patients are much more susceptible to infection with Plasmodium falciparum. Pregnant women have high malaria parasitemia in the placenta and more severe clinical disease, which affects not just the first pregnancy but all subsequent pregnancies. HIV-infected patients also do not respond as well to standard antimalaria treatment. Finally, malaria increases HIV viral load and raises the risk for mother-to-child HIV transmission (*29*). The biologic interaction between these diseases not only complicates treatment in an already challenging setting but also presents a serious risk for death to pregnant girls <19 years of age.

## Children Delivering Children

Births resulting from child marriages are said to be "too soon, too close, too many, or too late" (*33*). For example, a high percentage of girls in Ethiopia (25%), Uganda (42%), and Mali (45%) have given birth by the age of 18 compared with only 1% in Germany, 2% in France, and 10% in the United States (*1*). The problem with children delivering children is that the young mothers are at a significantly higher risk than older women for debilitating illness and even death. Compared with women >20 years of age, girls 10–14 years of age are 5–7 times more likely to die from childbirth, and girls 15–19 years of age are twice as likely (*34*). For example, in Mali, the maternal mortality rate for girls aged 15–19 is 178 per 100,000 live births and for women aged 20–34, only 32 per 100,000. In Togo, for the same age groups, these rates are 286 and 39, respectively (*1*). Reasons for these high death rates include eclampsia, postpartum hemorrhage, HIV infection, malaria, and obstructed labor. Obstructed labor is the result of a girl's pelvis being too small to deliver a fetus. The fetus's head passes into the vagina, but its shoulders cannot fit through the mother's pelvic bones. Without a cesarean section, the neonate dies, and the mother is fortunate if she survives. If sepsis or hemorrhage does not occur and the girl does survive, the tissue and bones of the neonate will eventually soften and the remains will pass through the vagina.

Many times, obstructed labor leads to fistulas; the pressure of the fetal head on the vaginal wall causes tissue necrosis, and fistulas develop between the vagina and the bladder or rectum after the necrotic tissue sloughs. More than 2 million adolescents are living with fistulas, and fistulas develop in ≈100,000 more each year (*35*). Girls ages 10–15 years are especially vulnerable because their pelvic bones are not ready for childbearing and delivery. Their risk for fistula is as high as 88% (*36*). Once a fistula is formed, fecal or urinary incontinence and peroneal nerve palsy may result and may lead to humiliation, ostracism, and resultant depression. Unless the fistula is surgically repaired, these girls have limited chances of living a normal life and bearing children.

## Effects on Offspring

Child marriage affects more than the young girls; the next generation is also at higher risk for illness and death. Adolescent mothers have a 35%–55% higher risk than older women for delivering infants who are preterm and of low birthweight. Mortality rates are 73% higher for infants born to mothers <20 years of age than for those born to older mothers (*37*). The infant mortality rates in Mali are 181 per 1,000 children born to women <20 years and 111 per 1,000 born to mothers ages 20–29 years; in Tanzania these rates are 164 and 88, respectively (*1*). These deaths may be partly because the young mothers are unhealthy, immature, and lack access to social and reproductive services. Their babies are also at high risk of acquiring HIV at delivery and during breastfeeding. Mothers who have had malaria are at increased risk for premature delivery, anemia, and death. Untreated STDs such as gonorrhea, chlamydia, syphilis, and herpes simplex virus infection can have deleterious effects on neonates, such as premature delivery, congenital neonatal infections, and blindness. Even the mortality rate for children <5 years can be 28% higher for children born to young mothers than for those born to mothers >20 years (*38*).

## Discussion

Child marriage has far-reaching health, social, economic, and political implications for the girl and her community. It truncates a girl's childhood, creates grave physical and psychological health risks, and robs her of internationally recognized human rights. Ending child marriage requires the consent of all those involved, including fathers and religious, community, and tribal leaders. To break the cycle of poverty, programs are needed to educate and empower women. In 2000, eight Millennium Development Goals outlined a vision that committed member countries to eradicate extreme poverty and hunger, educate all children through primary school, empower women, reduce childhood death, improve mothers' health, combat HIV/AIDS and malaria, ensure environmental sustainability, and develop a global partnership for development by the year 2015. Most of these goals directly affect child marriage. Data show that improvements are being made and that sub-Saharan Africa has the most obstacles to overcome (*39*).

In some countries, child marriage has been declining. Increasing mean age for marriage often results in part from overall advancement of an economy. In some countries, such as Korea, Taiwan, and Thailand, decreasing poverty effectively decreased child marriage by enabling these countries to improve education, increase employment, and provide better health care for the whole nation. Education is a key factor for delaying first sexual activity, pregnancy, marriage, and childbearing. Programs that specifically focused on the status of girls may have directly or indirectly reduced the number of child marriages. Successful programs have provided economic and educational opportunities to young women and their families by employing girls with the specific goal of delaying marriage (*40*), giving families financial incentives to keep their daughters in school (*1*), or feeding children during school to decrease families' expenses. Keeping girls in school or vocational training not only helps protect them from HIV infection, pregnancy, illness, and death but also enhances their earning potential and socioeconomic status. Educated girls can contribute to the health and welfare of their family and marry men of their own choosing and age.

Lack of enforcement renders laws against child marriage ineffective. Through media campaigns and educational outreach programs, governments need to take responsibility for stopping this practice. Local, regional, and national governments can also implement health outreach programs for girls and boys. Learning about reproductive and sexual health, STD prevention, contraception, AIDS, and how to seek health care helps girls negotiate safer sex. Governments must incorporate preventive and treatment programs for reproductive health issues into their health services. Necessary preventive services include supplying mosquito netting and condoms; educating patients about contraceptive methods; providing diagnostic screening for HIV and HPV; and offering treatment options such as medications, cesarean sections, and postpartum care.

Ending child marriage requires a multifaceted approach focused on the girls, their families, the community, and the government. Culturally appropriate programs that provide families and communities with education and reproductive health services can help stop child marriage, early pregnancies, and illness and death in young mothers and their children.
